# A unique assemblage of cosmopolitan freshwater bacteria and higher community diversity differentiate an urbanized estuary from oligotrophic Lake Michigan

**DOI:** 10.3389/fmicb.2015.01028

**Published:** 2015-09-29

**Authors:** Ryan J. Newton, Sandra L. McLellan

**Affiliations:** School of Freshwater Sciences, University of Wisconsin-MilwaukeeMilwaukee, WI, USA

**Keywords:** bacterial community, freshwater, urban ecology, Lake Michigan, oligotyping, bacterioplankton

## Abstract

Water quality is impacted significantly by urbanization. The delivery of increased nutrient loads to waterways is a primary characteristic of this land use change. Despite the recognized effects of nutrient loading on aquatic systems, the influence of urbanization on the bacterial community composition of these systems is not understood. We used massively-parallel sequencing of bacterial 16S rRNA genes to examine the bacterial assemblages in transect samples spanning the heavily urbanized estuary of Milwaukee, WI to the relatively un-impacted waters of Lake Michigan. With this approach, we found that genera and lineages common to freshwater lake epilimnia were common and abundant in both the high nutrient, urban-impacted waterways, and the low nutrient Lake Michigan. Although the two environments harbored many taxa in common, we identified a significant change in the community assemblage across the urban-influence gradient, and three distinct community features drove this change. First, we found the urban-influenced waterways harbored significantly greater bacterial richness and diversity than Lake Michigan (i.e., taxa augmentation). Second, we identified a shift in the relative abundance among common freshwater lineages, where acI, acTH1, *Algoriphagus* and LD12, had decreased representation and *Limnohabitans, Polynucleobacter*, and *Rhodobacter* had increased representation in the urban estuary. Third, by oligotyping 18 common freshwater genera/lineages, we found that oligotypes (highly resolved sequence clusters) within many of these genera/lineages had opposite preferences for the two environments. With these data, we suggest many of the defined cosmopolitan freshwater genera/lineages contain both oligotroph and more copiotroph species or populations, promoting the idea that within-genus lifestyle specialization, in addition to shifts in the dominance among core taxa and taxa augmentation, drive bacterial community change in urbanized waters.

## Introduction

As a result of continued urbanization worldwide and its contribution to deteriorating ecosystem services (Corvalan et al., 2005), the relationship between urban development, biodiversity patterns, and ecosystem dynamics has been the focus of increasing research attention and theoretical development (Grimm et al., 2000; Alberti, 2005; Pickett et al., 2011). In aquatic ecosystems, urbanization alters watershed ecosystem functioning through the movement, magnitude, and content of surface water runoff (Allan, 2004; Alberti et al., 2007; Hale et al., 2015). As a major component of aquatic biological communities, bacteria are critical drivers of energy flow and nutrient recycling (Cotner and Biddanda, 2002), yet we know relatively little about bacterial biodiversity patterns in urban-influenced waterways, whether there are important differences in the bacterial assemblages between urbanized and non-urbanized systems, or whether urban-influenced aquatic environments promote the persistence of organisms that impact human health or well-being (Paerl et al., 2003; Newton et al., 2013; King, 2014).

The effects of urban landscape modification can account for much of the water quality deterioration in urbanized waterways (Brabec et al., 2002), which characteristically have high solute (Booth and Jackson, 1997; Kaushal and Belt, 2012) and nutrient (Carpenter et al., 1998; Wollheim et al., 2005; Hale et al., 2015) loads and high productivity (Correll, 1998). Both the total productivity and the heterogeneity in nutrient resources play a prominent role in structuring species co-existence patterns across all scales of life (Mittelbach et al., 2001; Chase and Leibold, 2002; Jankowski et al., 2014). However, the mechanisms driving these compositional changes in response to increased ecosystem productivity are complex and at minimum depend upon the total resource pool, the balance of resources within this pool, and the richness of competing species for specific resources (Cardinale et al., 2009). Since urbanization results in increased delivery of nutrients to surface waters (Carpenter et al., 1998; Paul and Meyer, 2001), high nutrient concentration is likely one driver of changes in the bacterial assemblage in these systems. For this reason, the patterns of bacterial community assembly across an urbanization gradient may in large part mirror those observed across trophy or primary productivity gradients.

Increased productivity or nutrient load has been shown to relate to changes in the diversity and composition of bacterial communities in freshwater ecosystems (Horner-Devine et al., 2003; Yannarell and Triplett, 2004; Longmuir et al., 2007; Smith, 2007; Kolmonen et al., 2011; Jankowski et al., 2014). Yet a clear relationship between productivity and bacterial diversity or community change has not been identified consistently. For example, bacterial richness was uncoupled to total phosphorus concentration in 100 lakes in Finland (Korhonen et al., 2011) and productivity related variables were not strong predictors of community composition across 30 lakes in Wisconsin, USA when geographic and landscape related variables were considered (Yannarell and Triplett, 2005). Also, several processes have been implicated in driving bacterial community change across aquatic environmental gradients, including: complete community displacement or turnover (Bell et al., 2010), changes in the relative abundance of a few core taxa (Shade et al., 2010), and an increase in the presence of rare or novel taxa that augment a core community (Jankowski et al., 2014; Shade et al., 2014). These varied and sometimes contradictory findings suggest that the relationship between microbial community structure and ecosystem productivity are complex and still poorly defined.

Few studies that examined explicitly the relationship of system productivity and bacterial community change also identified the bacterial types causing the observed change. In one such study, an increased representation of rare and/or novel taxa in more eutrophic conditions were implicated as being responsible for much of the observed community change, but the taxonomic affiliation of these taxa were not considered (Jankowski et al., 2014). Studies involving the distribution and growth traits of common lake taxa have provided some insight into which taxa would be expected to drive changes across productivity/trophy gradients. Specifically, members of the genus *Limnohabitans* and *Flavobacterium* exhibited high maximum growth rates and abundance correlations to high nutrient conditions in lakes (Šimek et al., 2006; Newton et al., 2011a; Neuenschwander et al., 2015), while the freshwater lineages LD12 and acI have slower growth rates and traits indicating a more oligotrophic lifestyle (Šimek et al., 2006; Newton et al., 2011a; Salcher et al., 2011b; Ghylin et al., 2014).

Using an analysis of bacterial community composition along sample transects from the highly urbanized waterways in the Milwaukee estuary to the relatively low urban-impacted waters of Lake Michigan, we assess how the bacterial assemblage differs between these two connected environments. Specifically we evaluate whether processes identified as driving microbial community change in aquatic systems, such as complete community turnover, shifts in the community contribution of common taxa, or taxa augmentation also drive changes in the richness and composition of bacteria across an urbanization gradient. With these data we also identify the taxa responsible for differences in the community assemblages across the urban-influence gradient and evaluate whether there are differential distribution patterns for narrowly-defined sequence-based groups (oligotypes) within several ubiquitous freshwater genera/lineages.

## Materials and methods

### Sample collection and site characteristics

**Figure 1:**
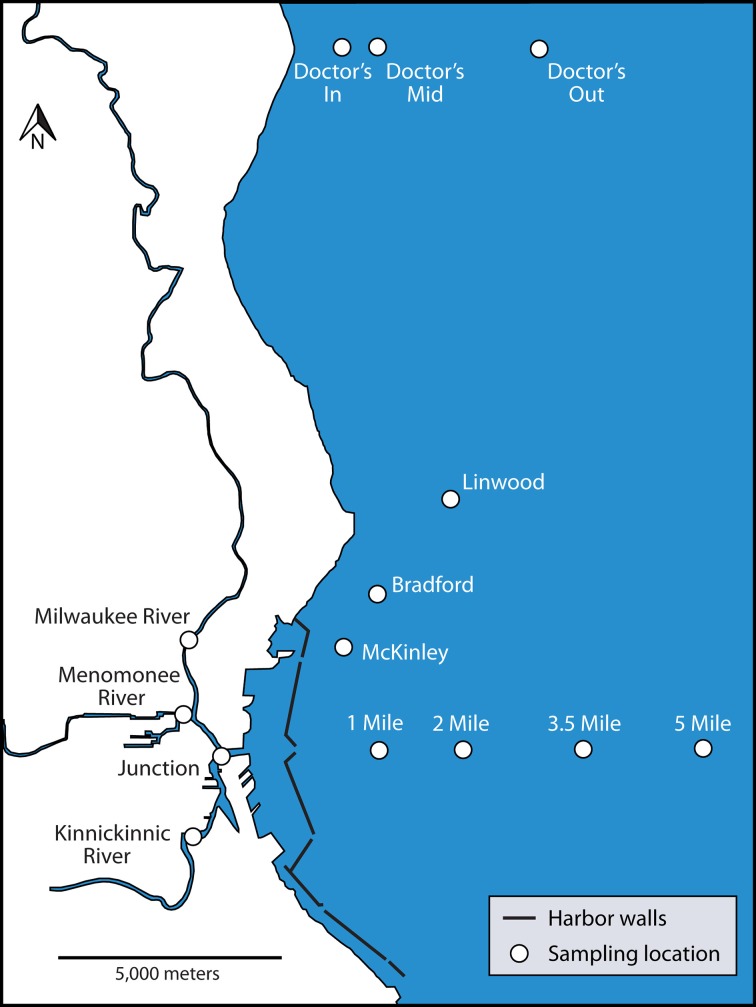
**Map of Milwaukee, WI, USA urban estuary and nearshore Lake Michigan**. Sampling locations included in this study are indicated with site names.

All samples analyzed for bacterial community composition were collected from surface waters (0–0.5 m depth) during the ice-off season (April to October) in the waterways of Milwaukee, WI or in Lake Michigan. Each final sample consisted of three surface water samples that were combined, mixed, and subsampled into 1- to 4-l bottles. The samples were collected on 15 separate expeditions spanning the years 2008–2012. See Figure 1 for a sample map of the collection locations and Supplementary Table 1 for sample metadata. Samples collected in 2008–2010 were described previously (Newton et al., 2013). Sample processing and filtering methods are described in Newton et al. (2011b).

We characterized the average or “typical” chemical and physical conditions of the waterways using data from the Milwaukee Metropolitan Sewerage District Water Quality Monitoring program housed via the WATERBase database at the University of Wisconsin-Milwaukee (www.waterbase.glwi.uwm.edu/). From these data, we retrieved surface water sample measurements collected on 19 occasions for Lake Michigan and on 31 occasions for the rivers and inner harbor. These samples were limited to the months of June through October for the years 2008–2010, which represents a similar seasonal period and most of the years during which the bacterial community water samples were obtained. Three sample sites (2 mile, Linwood, and Doctors Out) were used to represent Lake Michigan and one sample site each was used to represent each of the rivers and the inner harbor (see Figure 1 for sample locations). Data was obtained for water temperature, pH, conductivity, suspended solids, total phosphorus, Total Kjeldahl Nitrogen, nitrate/nitrite, and chlorophyll *a* according to the standard protocols listed in the Standard Methods for the Examination of Water and Wastewater (20th ed., 1998)^1^. The median and range for each environmental parameter at each sample site are listed in Table 1.

**Table 1:** **Chemical and physical properties of sampled environments^a^^,^^b^^,^^c^**.

	**Urban estuary**				**Lake Michigan**		
	**Junction**	**MKE**	**MN**	**KK**	**2 mile**	**Linwood**	**Doctors park out**
Temperature (°C)	19.4 (11.8–24.6)	21.6 (7.5–26.8)	22.1 (12.7–29.1)	19.3 (12.3–25.0)	17.0 (10.4–21.7)	17.5 (8.6–21.5)	16.4 (6.9–22.9)
pH	8.0 (7.5–8.5)	8.2 (7.7–8.6)	7.8 (7.4–8.3)	8.0 (7.6–8.4)	8.4 (8.0–8.7)	8.4 (8.2–8.6)	8.4 (8.2–8.6)
Conductivity (μS/cm)	587 (257–799)	807 (210–896)	700 (336–997)	615 (315–899)	294 (279–341)	285 (274–305)	285 (275–292)
Susp. solids (mg l^−1^)	6 (4–80)	12 (4–100)	8 (3–170)	10 (6–140)	bd (bd–bd)	bd (bd–bd)	bd (bd–bd)
Total P (μ*gl*^−1^)	68 (bd–230)	115 (44–290)	100 (bd–280)	82 (42–260)	bd (bd–72)	bd (bd–bd)	bd (bd–25)
TKN (mg l^−1^)	0.60 (bd–1.10)	0.72 (bd–1.60)	0.68 (bd–1.60)	0.62 (bd–1.40)	bd (bd–0.62)	bd (bd–0.83)	bd (bd–0.84)
NO_3_/NO_2_ (mg l^−1^)	0.71 (0.38–1.10)	0.90 (0.24–1.40)	0.58 (0.27–1.10)	0.71 (0.35–1.20)	0.27 (bd–0.44)	0.26 (bd–0.30)	0.29 (bd–0.31)
Chlorophyll *a* (μ*gl*^−1^)	6.0 (0.9–17.8)	6.4 (3.3–34.8)	6.7 (2.1–52.9)	6.0 (1.8–27)	1.2 (0.3–7.2)	0.8 (bd–7.5)	0.4 (0.2–1.8)

a The Median (Range) are listed for each water chemical/physical property measurement.

b Abbreviations: Susp. Solids, Suspended Solids; Total P, Total Phosphorus; TKN, Total Kjeldahl Nitrogen; bd, below detection.

c The detection limits are as follows: Suspended Solids 1 mg l^−1^, Total Phosphorus 20 μg l^−1^, Total Kjeldahl Nitrogen 0.36 mg l^−1^, Nitrate/Nitrite 0.20 mg l^−1^, Chlorophyll a 0.11 μg l^−1^.

Based on the environmental parameters representing each area and the connection between each waterway to the urban landscape, we grouped the sample locations into two categories: (1) urban-impacted and (2) Lake Michigan, respectively representing high and low impact from urban discharge. The urban-impacted category includes the three rivers and inner harbor samples and the Lake Michigan category includes all samples outside of the harbor break walls (see Figure 1 for sample locations).

### 16S rRNA gene sequencing and processing

DNA extraction procedures for all filtered water samples are detailed in Newton et al. (2013). Extracted DNA was used to construct amplicon libraries for high-throughput 16S rRNA gene sequencing targeting either the V6 or V4 to V6 regions (amplified in the reverse direction V6 to V4). Amplicon libraries were sequenced using either the 454 Life Sciences or the illumina® platform. Details for amplicon library construction, sequencing procedures, and post-sequencing quality control methods for the V6 454 platform are described in McLellan et al. (2010), for the V6V4 454 platform in Newton et al. (2013), and for the illumina® V6 platform in Eren et al. (2013b). Sequencing methods for each sample are listed in Supplementary Table 1.

The National Center for Biotechnology Information Sequence Read Archive has archived the raw data under SRA Projects SRP018584 (V6 454), SRP059202 (V6V4 454), and SRP056973 (V6 illumina). Trimmed and quality filtered sequence data are publicly available from the Visualization and Analysis of Microbial Population Structures website (VAMPS; http://vamps.mbl.edu; Huse et al., 2014) under project names SLM_SWG_Bv6, SLM_NIH_Bv6v4, and SLM_NIH2_Bv6.

### Dataset construction

We used the algorithm Global Alignment for Sequence Taxonomy (GAST; Huse et al., 2008) to assign taxonomy to all sequences. A dataset consisting of sequences binned by the most resolved taxonomic assignment down to genus was used in whole community composition comparisons among samples. Analyses using this dataset are termed “taxon-based.” We also constructed a second, higher resolution dataset based on closed-reference clustering, where reads are searched against the curated SILVA database (Pruesse et al., 2007) as part of the Visualization and Analysis of Microbial Population Structures (VAMPS; http://vamps.mbl.edu) database (Huse et al., 2014) and then clustered as defined by the best database match for each read (see Huse et al., 2008 for more details). Since reference sequence matches are not identical across sequence regions (V6 vs. V6V4 data), but reference-based clustering provides more narrowly-defined groupings than taxon-based assignments, and therefore a more accurate representation of total bacterial diversity, this dataset was used only for richness and diversity comparisons. Analyses using this dataset are termed “reference-based.”

We constructed a third, high-resolution dataset to explore distribution patterns within and among common freshwater genera/lineages. This dataset consisted only of amplicons assigned by GAST to the *Actinobacteria* family *Sporichthyaceae* and genus *Aquiluna*, the *Bacteroidetes* genera *Algoriphagus, Arcicella, Flavobacterium, Fluviicola*, and *Sediminibacterium*, the *Proteobacteria* lineage SAR11 and genera *Hydrogenophaga, Polynucleobacter, Rhodobacter, Rhodoferax, Sphingopyxis*, and the *Verrucomicrobia* genus *Luteolibacter*. All amplicons assigned to these 14 common freshwater groups were aligned (within-group alignments) using the align.seqs command in mothur (Schloss et al., 2009). After alignment, the non-overlapping sequence from the V6V4 amplicons was trimmed from the 14 alignments using the filter.seqs command in mothur (Schloss et al., 2009). We then conducted a high-resolution oligotyping analysis on the trimmed alignments as described previously (Eren et al., 2013a; oligotyping.org). Oligotyping is a supervised computational method that uses Shannon entropy calculations to identify nucleotide variation in alignments. The entropy calculations are used to select highly variable positions in the alignment, which are then used to parse the data into groups having identical sequences at the defined positions. These highly-resolved groups are known as oligotypes (Eren et al., 2013a). We set the minimum substantive abundance criterion (*M*) to the lesser of 0.01% of all sequences assigned to each group or 10 and the minimum sample prevalence (*s*) to 2 for all 14 oligotyping analyses. Oligotypes were deemed to have converged when entropy values within each oligotype were below 0.2 according to the procedures described in Eren et al. (2013a).

For the family *Sporicthyaceae*, reference sequences from each oligotype were compared against the freshwater database from Newton et al. (2011a) to assign a more refined freshwater naming structure. *Sporichthyaceae* oligotypes were resolved to the lineages acI-A, acI-B, acI-C, acSTL, and acTH1 when the representative sequence was identical to or contained a single mismatch to sequences representing only one of the lineages. After splitting the *Sporichthyaceae* into five distinct lineages, our final oligotyping dataset consisted of 18 unique lineages that were used in subsequent analyses. For *Rhodoferax*, reference sequences for each oligotype were also compared against the Newton et al. (2011a) freshwater database and only those sequences identical to or with a single mismatch to sequences representing the *Limnohabitans* lineage were retained. The SAR11 GAST assigned sequences, throughout are referred to as LD12, the freshwater lineage to which these sequences belong.

Data on the distribution of freshwater taxa generated from clone library sequence data as reported in Newton et al. (2011a) were used in a community composition comparative analysis. These data include the relative abundance of common freshwater genera and lineages from the epilimnion of 47 lakes located primarily in North America and Europe, but also including Antarctica, Africa, and China. This database included only studies with data generated from universal bacterial primers and random clone selection for sequencing and for which more than 40 sequences were present (see Newton et al., 2011a for further dataset details).

### Statistical analyses

We conducted all data analyses in the R statistical language (R Core Team, 2013). We used the community analysis package *vegan* (Oksanen et al., 2013) and the Bray-Curtis dissimilarity metric for all community composition comparisons. Non-metric multidimensional scaling (NMDS) and hierarchical clustering were based on Bray-Curtis dissimilarities using the relative abundance of taxon- or reference-based groups, calculated as the sequence count for a group divided by the total sequence counts for a sample (whole community) or the sequence counts for a subset of taxa/lineages from a sample (e.g., common freshwater genera/lineages only). To identify the number of dimensions to include in NMDS analyses, a scree plot was used to identify dimensional convergence for ordination stress and a low dimension analysis (*k* = 2) was compared to a higher dimensionality analysis (*k* = 10) for significant ordination correlation using a Procrustes rotation via the *protest* function. Analysis of Similarity (ANOSIM) statistics (999 permutations) were carried out with the *anosim* function (Oksanen et al., 2013) and were used to test the significance of *a priori* assigned sample group differentiation. We used the Mann-Whitney *U*-test to examine whether the distribution of measurements for two groups differed significantly (Mann and Whitney, 1947). For most data visualization we used the *ggplot2* R package (Wickham, 2009) or base graphics in R. We constructed heatmaps with the *heatmap.2* function in the *gplots* R package (Warnes et al., 2013).

We used two measures of diversity, inverse Simpson index (Lande, 1996) and the tail statistic (Li et al., 2012) to compare among sample groups. These two metrics differ in their weighting of abundant vs. rare members in a sample. The inverse Simpson metric places more emphasis on the diversity of the most abundant taxa/groups among samples, while the tail statistic places more emphasis on the diversity of more rare community members (Li et al., 2012). We carried out inverse Simpson diversity calculations using the *diversity* function in the *vegan* package (Oksanen et al., 2013) and the tail statistic according to the equation developed by Li et al. (2012).

For all richness and diversity calculations and data comparisons using oligotypes of the common freshwater genera/lineages, we used a subsampled dataset to reduce the artifacts of disproportionate sequencing depth when using non-relativized data. We subsampled randomly once all samples having >30,000 quality-filtered sequences to 30,000 sequences using the R package *plyr* (Wickham, 2011; see Supplementary Table 1 for sequence read counts after subsampling).

To compare the magnitude of a “habitat preference” between the urban estuary waters and Lake Michigan for common freshwater genera/lineages, we used the ratio of the mean relative abundance of each genus/lineage among the urban estuary samples vs. its mean relative abundance in the Lake Michigan samples. To minimize the effect caused by differences in the proportion that these common freshwater bacteria make up in each sample, each genus/lineage relative abundance was calculated as the proportion of sequences in the high-resolution dataset of 18 common freshwater genera/lineages. To minimize the impact of temporal abundance variability for an individual genus/lineage, the relative abundance of each genus/lineage was normalized to the sample with the highest relative abundance within each sample transect.

To identify individual oligotypes that preferentially associated with either the urban-influenced waterways or Lake Michigan, we performed a multinomial species classification using the CLAM test (Chazdon et al., 2011) in the *vegan* R package (Oksanen et al., 2013). This model allowed us to divide oligotypes into the following four categories based on their distribution among samples: oligotypes preferential to urban-influenced waterways, oligotypes preferential to Lake Michigan, oligotypes showing no preferential distribution (generalists), and oligotypes that were too rare to classify with confidence. The CLAM test was performed on the subsampled dataset using an alpha value of 0.01 divided by the total number of oligotypes (*n* = 351), a coverage limit of 30, and a specialization threshold of 0.75. A specialization threshold = 0.67 (a supermajority) is considered conservative (Chazdon et al., 2011).

## Results

### The lake michigan bacterial community resembles other freshwater lakes but differs from milwaukee's urban-impacted waterways

**Figure 2:**
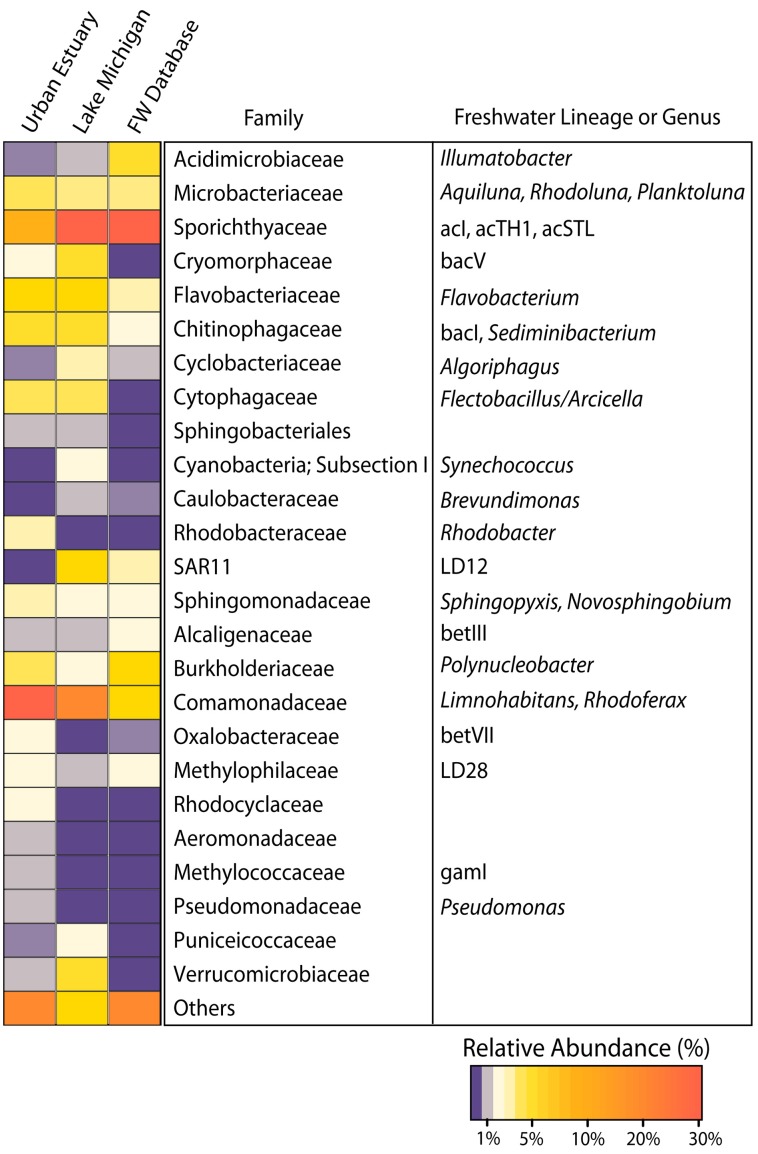
**Heatmap indicating the relative abundance of all bacterial families with a mean relative abundance of ≥1% among either all Urban Estuary or all Lake Michigan samples**. Common freshwater lineages as described in Newton et al. (2011a) are indicated with their respective family assignments. A previously compiled freshwater dataset is also depicted (FW Database) and consists of bacterial group distributions inferred from whole community 16S rRNA gene amplification and clone library construction across 47 lakes as described in Newton et al. (2011a).

The surface water community in relatively nearshore (< 10 km from shore) Lake Michigan is dominated by many of the freshwater bacterial genera and lineages that are common to the surface waters of smaller freshwater lakes (Figure 2). On average, the bacterial families in Lake Michigan with the highest number of assigned sequence reads were *Sporichthyaceae*, (28%; freshwater lineages acI, acTH1, and acSTL), *Comamonadaceae*, (13%; freshwater genera *Limnohabitans and Rhodoferax*), *Flavobacteriaceae* (8%; freshwater genera *Flavobacterium*), SAR11 (7%; freshwater lineage LD12), and *Verrucomicrobiaceae* (5%). The families *Sporichthyaceae, Comamonadaceae, and Flavobacteriaceae* were the only bacterial families that averaged ≥5% of the reads in samples from the urban-impacted waterways. In addition to these common freshwater lineages, the urban impacted waterways also harbored other bacterial families at relatively high abundances (each at ≥2% of the community) that were not common in Lake Michigan, namely, *Oxalobacteraceae* (freshwater lineage betVII), *Rhodocyclaceae*, and *Rhodobacteraceae* (freshwater genera *Rhodobacter*).

**Figure 3:**
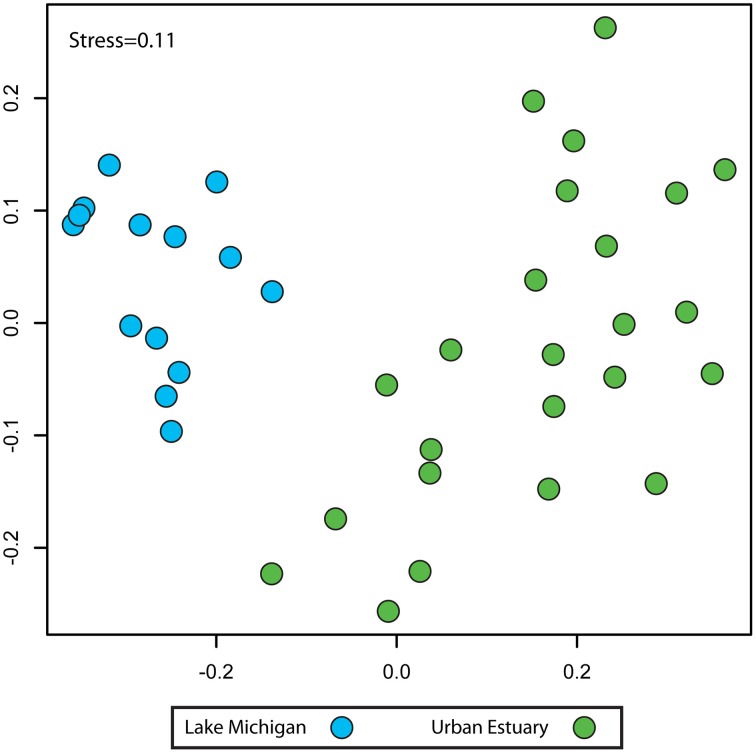
**Non-metric multidimensional scaling plot indicating the community composition relationships (Bray-Curtis similarity) between the Urban Estuary (green) and Lake Michigan (blue) samples**. Community composition is based on the grouping of sequences by taxonomic assignment to genus and compiled as the relative contribution of each taxon to the community.

NMDS analysis of sequence data binned by taxonomic assignment to genus (taxon-based) indicated the urban-impacted water (rivers and inner harbor) communities were distinct from the bacterial communities of Lake Michigan (Figure 3; urban-impacted vs. Lake Michigan; ANOISM *R* = 0.80 *p* = 0.001). Since three different sequencing region/platform combinations were used to create these data, we examined whether this community composition pattern was influenced by the sequencing procedures used (see Supplementary Table 1 for sample details). We found there was a significant, but small proportion of the community variation explained by sequencing procedure (ANOSIM *R* = 0.15, *p* = 0.009), and this variation was distinct from and much smaller than the variation separating the urban-water and Lake Michigan communities (Supplementary Figure 1). Two dimensions were used in the final NMDS ordination calculation, as ordination stress was relatively low (0.11) and additional dimensions did not alter the sample relationship patterns observed (Procrustes test for ordination similarity between *k* = 2 and *k* = 10; *r* = 0.801, *p* = 0.001).

### Taxa augmentation in urban waterways

The microbial communities present in the urban waters had higher taxonomic (taxon-based, binned by genus assignment) and reference-based (binned by reference sequence) richness than the communities from Lake Michigan (Table 2). The urban water communities also contained higher alpha-diversity levels than the Lake Michigan communities, and this diversity increase was observed with both the inverse Simpson index (reference-based analysis) and the tail statistic (taxon- and reference-based; Table 2). Only the taxon-based diversity comparison, using the inverse Simpson test, showed no significant difference between the urban-impacted water communities and Lake Michigan (*p*>0.01; Table 2).

**Table 2:** **Diversity comparison between Urban estuary and Lake Michigan samples^*a*^**.

	**Taxon—Whole community**			**Reference sequence—Whole community**		
**Sample environment**	**Richness**	**Inverse simpson**	**Tail**	**Richness**	**Inverse simpson**	**Tail**
Urban estuary	432 ± 104	7 ± 6	49 ± 23	2680 ± 1232	68 ± 40	584 ± 463
Lake Michigan	185 ± 25	5 ± 2	16 ± 3	1015 ± 440	37 ± 14	145 ± 85
Mann-Whitney U	400^**^	277^*^	400^**^	379^**^	325^**^	370^**^

a Mean and standard deviation are reported.

** Indicates significance at p < 0.01.

* Indicates significance at p < 0.05.

Most of the identified taxa in Lake Michigan were also detected in the urban-impacted waters. For example, of the 1458 taxa identified in at least two samples, only one was present solely in Lake Michigan, while 397 were present solely in the urban-impacted waterway samples. However, these 397 urban-water associated taxa did not typically comprise a large part of the community, contributing on average only 0.14% of the sequence reads in the urban-waterway samples. Together these data indicate an increased distinction between the urban waterways and Lake Michigan as the grouping method used to identify organisms becomes more narrow (from taxon- to reference-based) and as the diversity index puts more weight on more rare organisms (from inverse Simpson to Tail), suggesting a higher number of more closely related (within-genus), but relatively rare organisms in the urban waterways.

### Common freshwater taxa exhibit differential preference for urban-impacted vs. lake michigan waters

After examining the whole bacterial community composition differences between the urban-impacted and Lake Michigan waters, we further examined the distribution of 18 common freshwater genera/lineages across four sampling transects. The 18 genera/lineages included: acI-A, acI-B, acI-C, acTH1, acSTL, *Aquiluna, Algoriphagus, Arcicella, Flavobacterium, Fluviicola, Sediminibacterium*, LD12, *Sphingopyxis, Hydrogenophaga, Limnnohabitans, Polynucleobacter, Rhodobacter*, and *Luteolibacter*. All genera/lineages were present in all samples (*n* = 23) except for LD12 (22/23) and acI-C (21/23). These 18 genera/lineages comprised on average 44.9 ± 6.9% of the sequence reads in the urban water communities and on average 69.3 ± 2.6% of the Lake Michigan communities.

**Figure 4:**
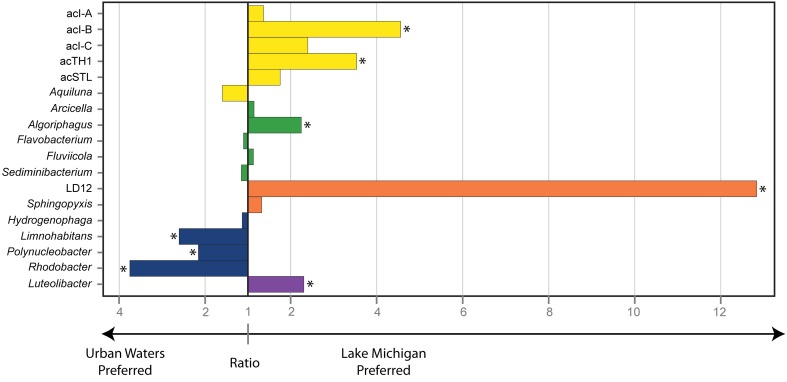
**Magnitude of habitat preference between the Urban Estuary waters and Lake Michigan for common freshwater genera/lineages**. Habitat preference is determined by the ratio of the mean relative abundance of each genus/lineage among the urban estuary samples vs. its mean relative abundance in the Lake Michigan samples. Bars plotting to the left indicate an urban estuary preference while bars plotting to the right indicate a Lake Michigan preference. A significant association (Mann-Whitney *U*-test, *p* ≤ 0.01) with either environment is indicated by an asterisk. Bar color indicates bacterial phylum where yellow, *Actinobacteria*; green, *Bacteroidetes*; orange, *Alphaproteobacteria*; blue, *Betaproteobacteria*; and purple, *Verrucomicrobia*.

The relative abundance of the 18 common freshwater lake bacteria genera/lineages (calculated as relative to each other) indicated differential distributions in the urban-impacted waters vs. Lake Michigan (ANOSIM *R* = 0.65 *p* < 0.001), suggesting some common lineages were favored over the others by the conditions present in each environment. We explored whether individual genera/lineages exhibited a “preference,” defined as an increased average relative abundance vs. the other common genera/lineages, for either the urban impacted or non-impacted Lake Michigan waters. We found that some genera/lineages were favored by the conditions present in the urban waterways, while others were favored by the conditions in Lake Michigan (Figure 4). The organisms affiliated with the *Actinobacteria* lineages acI-B, acI-C, and acTH1 the *Alphaproteobacteria* lineage LD12, and the *Cytophagia* genus *Algoriphagus* had a strong preference for the conditions in Lake Michigan, while the *Betaproteobacteria* genera *Rhodobacter, Polynucleobacter*, and *Limnohabitans* had a strong preference for the urban-impacted waters (Figure 4).

### Oligotyping reveals unique environmental distribution patterns within common freshwater lake taxa

We used oligotyping to provide a refined sequence-based analysis for each the 18 common freshwater lake genera/lineages (see Materials and Methods for details). The 18 genera/lineages were represented by 351 oligotypes. In contrast to the whole community, the common freshwater lake genera/lineages did not exhibit significant richness or diversity differences (*p*>0.01) between the urban-impacted and Lake Michigan waters (Table 3). These data in conjunction with the whole community diversity differences indicate that a similar level of diversity for common lake bacteria is present across both environments, but in the urban-impacted waterways these common lake community members are augmented with a large number of microorganisms that are uncommon in lake surface waters.

**Table 3:** **Oligotype diversity comparisons for common freshwater genera/lineages^a^**.

**Sample environment**	**Oligotype—Core freshwater**		
	**Richness**	**Inverse simpson**	**Tail**
Urban estuary	144 ± 20	14 ± 4	19 ± 4
Lake Michigan	127 ± 18	14 ± 4	16 ± 2
Mann-Whitney U	92	64	96^*^

a Mean and standard deviation are reported.

* Indicates significance at p < 0.05.

**Figure 5:**
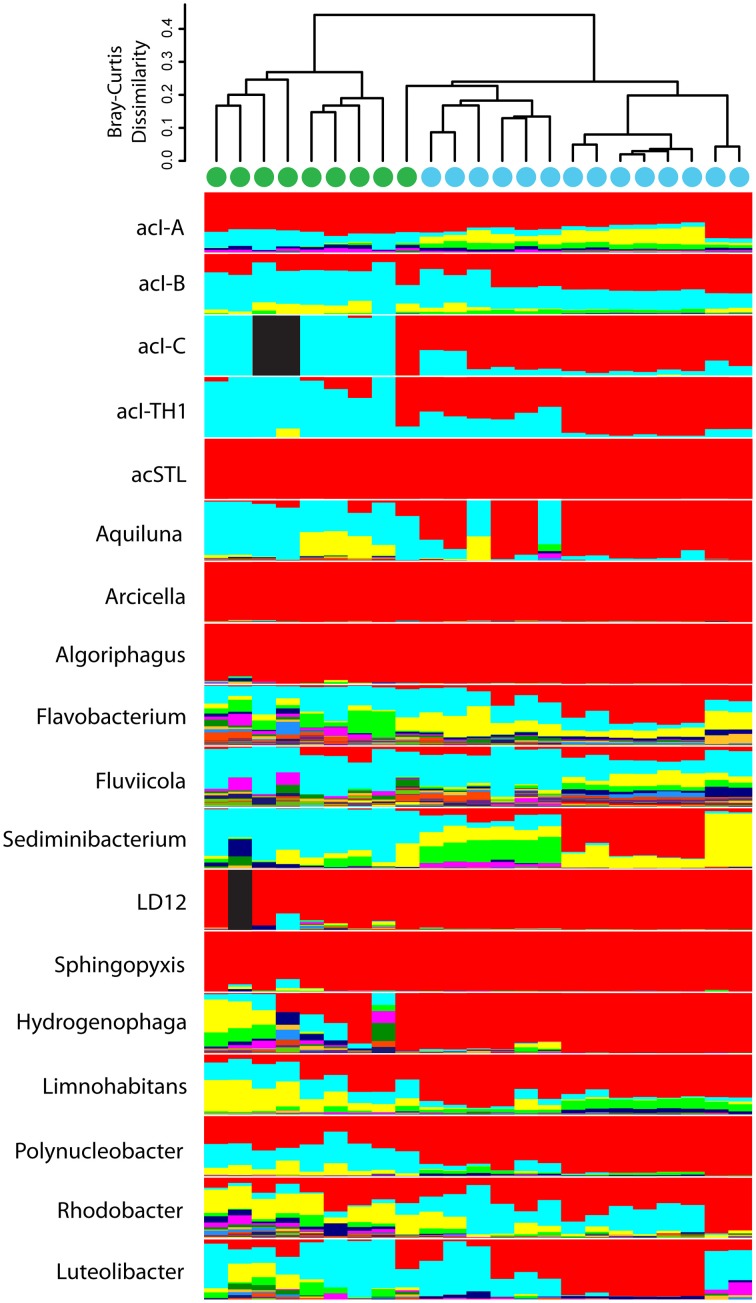
**Oligotype composition within each freshwater bacterial genus/lineage among samples is indicated in stacked bar plots**. The relationship of the oligotype composition in each sample is depicted with a cluster dendrogram based on Bray-Curtis dissimilarity among samples. Fully black bars indicate no sequences were recovered from that genus/lineage in that sample. Samples were clustered via an unweighted pair group method with arithmetic mean calculation. Samples collected from the urban estuary are labeled with a green circle while those collected from Lake Michigan are labeled with a blue circle.

**Figure 6:**
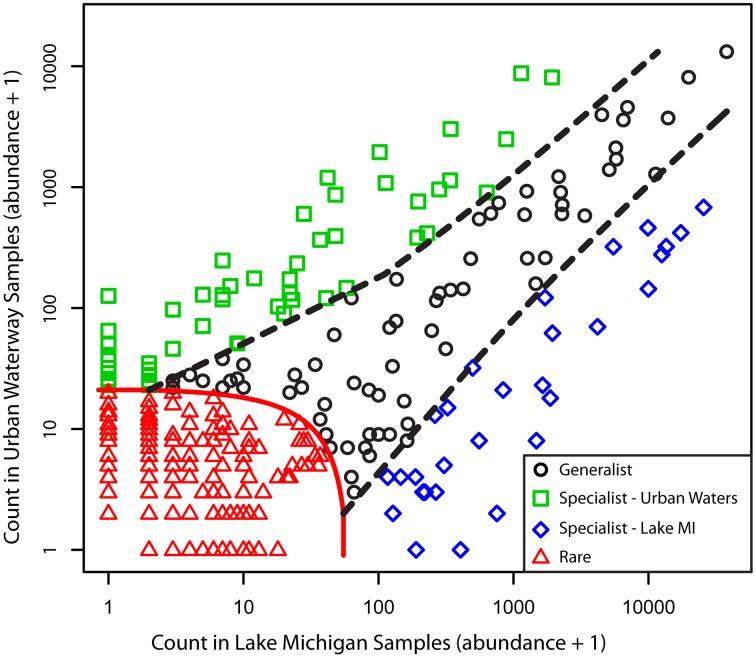
**Classification results for oligotype distributions between the urban estuary and Lake Michigan samples**. Specialization was set with a threshold of *K* = 0.75, a coverage limit = 30, and *P* = 0.01/351. Classification status is indicated by point color and shape. The specialist and rarity thresholds are indicated by dotted lines.

Although the common freshwater genera/lineages oligotype richness and diversity did not differ significantly between the urban-impacted and Lake Michigan samples, there was a significant difference in the distribution of these oligotypes between the two environments (Figure 5; ANOSIM *R* = 0.90, *p* < 0.001). A CLAM statistical approach using stringent conditions for environmental specialist determination (see Materials and Methods) indicated 80 of the 351 oligotypes exhibited significantly differentiated distributions between the two environments (51 associated with urban waters and 29 with Lake Michigan; Figure 6). The *Actinobacteria* lineages (acIA, acIB, acIC, acTH1) and the genus *Fluviicola* harbored the majority of Lake Michigan favored oligotypes (20; Supplementary Table 2), while the genera *Flavobacterium, Hydrogenophaga, Limnohabitans*, and *Rhodobacter* harbored a large number of the urban-water favored oligotypes (36; Supplementary Table 2). In several cases, oligotype pairs with one or two nucleotide differences (>97 or >96% identity, respectively) had opposite preferences for the urban waters vs. Lake Michigan (Supplementary Table 2).

## Discussion

We observed a strong division between the bacterial community composition in the urban-impacted waterways of the Milwaukee estuary and of those in greater Lake Michigan. This result was not surprising given the numerous differences in the chemical and physical conditions of these two distinct but connected systems. In particular, higher nutrient and particle loads, water temperature, and lower residence time differentiate the sampled urban estuary waters and the waters of oligotrophic Lake Michigan. Nutrient and particle load, residence time, and temperature are all parameters that have been shown to impact the bacterial community makeup of freshwater systems (Lindström et al., 2005; Allgaier and Grossart, 2006; Newton et al., 2011a). Here we did not attempt to distinguish among these parameters as a driving force for community differentiation. Instead, we sought to further our understanding of urban influences on aquatic bacterial communities by identifying how the bacterial assemblages of urbanized waterways differed from those of a connected, but oligotrophic low urban-impacted system. Our study shows that a core pelagic bacterial community is present across this urban-eutrophic to oligotrophic gradient, as at all levels of classification: (1) taxon—genus, 2) sequence—reference-based, and (3) oligotype, the majority of sequence types in the lake were also recovered from the urban estuary. However, large changes in the bacterial assemblages were also present, notably a loss of diversity among taxa/lineages not considered common to lakes during the transition from the estuary to the open lake and a significant composition change both among cosmopolitan freshwater taxa/lineages and for oligotypes within these taxa/lineages.

### Taxa augmentation in urban-influenced waters

Our results showed that bacterial richness was higher in the urban waterways, supporting what had been reported in several studies examining bacterial community trends across lake productivity/trophy gradients (Kolmonen et al., 2011; Logue et al., 2012; Jankowski et al., 2014). The bacterial diversity estimates that emphasized more rare community members resulted in a larger diversity disparity between the urban estuary and Lake Michigan habitats, indicating the presence of a much larger pool of rare community members in the urban-influenced waterways. Our diversity estimates also indicated this rare pool of organisms was not derived from genotypic variation within the most common freshwater genera/lineages as at our finest scale of organism resolution, the oligotype, there were not on average differences in the richness and diversity between the two environments. Instead, we suggest a typical pelagic freshwater community in the urban estuary was being augmented by a large number of more rare freshwater organisms and/or organisms not found in pelagic lake communities.

In a lake productivity gradient study, Jankowski and coauthors suggested that increased habitat heterogeneity, which is typically associated with higher nutrient lake systems, provides additional resources that allow rare or absent taxa in oligotrophic systems to flourish in more eutrophic waters (Jankowski et al., 2014). Although we did not examine habitat heterogeneity explicitly here, it is likely a contributing factor to the increased diversity in the urban-influenced waterways. In our system, the variation, which is one measure of habitat heterogeneity, in the chemical and physical characteristics of the urban-influenced waters was much higher than that in greater Lake Michigan (Table 1). In support of the relationship between high habitat heterogeneity and recruitment of otherwise rare freshwater taxa, all but one taxon (categorized to genus) present in Lake Michigan was also present in at least one urban estuary sample, but nearly 400 taxa were detected only in the urban estuary samples. Also the larger number of oligotypes (51 vs. 29) from the common freshwater genera/lineages that were classified as being “urban-water” vs. “Lake Michigan” specialists may be a reflection of the increased resource diversity in the urban estuary.

It is also likely that surface runoff and stormwater discharge contributed significantly to the increased diversity observed in the urban-impacted waters. Impervious surfaces decrease water infiltration and increase surface runoff, and storm sewers redirect normal water flow. Together, these urban constructions dramatically alter the flow of water into urban surface waters (Brabec et al., 2002; Kaushal and Belt, 2012). In previous work, we estimated that under typical weather conditions, 2–11% of the 16S rRNA genes recovered in Milwaukee estuary samples had an urban environment origin (Fisher et al., 2015). We also found that some of these organisms, including organisms indicating human fecal pollution, were present consistently in the estuary over several years of sampling (Newton et al., 2011b; Fisher et al., 2014). At this time, it is not clear whether these organisms persist because dispersal is frequent enough from the urban environment to overcome local environmental dynamics (i.e., mass effects) or whether the conditions in these urban waterways allow these organisms to have prolonged survival and/or grow (i.e., species sorting; Lindström and Langenheder, 2012). If pathogenic organisms are maintained or proliferate in urban water systems, then these waterways may present a greater human health risk than previously recognized (Fisher et al., 2014). Our data certainly suggest that the delivery of a large number of foreign, “urban-derived” bacteria may be common in urbanized waterways. This potentially massive immigration combined with the increased habitat heterogeneity in more eutrophic systems, appears to create a significantly more diverse bacterial assemblage in urbanized systems. We also note these data further support the idea that bacterial community assemblage patterns across productivity gradients contrast those for other organisms like fish and zooplankton, which typically exhibit decreased diversity in high productivity systems (Dodson et al., 2000; Barnett and Beisner, 2007; Jankowski et al., 2014).

### Core freshwater community shifts

Although we observed differences in the bacterial community composition between the urban estuary and Lake Michigan environments, the whole community analysis approach was not sufficient to identify whether these differences were the result of increased diversity in the urban-influenced waterways or stemmed from a combination of changes among rare and common organisms. Previous work across lake trophic gradients suggests that some bacterial groups are widespread (Jezbera et al., 2011, 2013; Kolmonen et al., 2011; Newton et al., 2011a; Jankowski et al., 2014), which could indicate most of the changes in eutrophic communities result from the increased abundance of rare or absent organisms in oligotrophic systems. Indeed changes in the so-called “conditionally rare taxa” can be a dominant driver of community change across environmental gradients (Shade et al., 2014). However, shifts in the dominant or common community members also frequently drive change in the bacterial community composition across environmental gradients (e.g., Gobet et al., 2010; Shade et al., 2010).

We used 18 ubiquitous freshwater lake genera/lineages to compare change in the composition among dominant freshwater taxa. Although these genera/lineages comprised a large proportion of the community in both environments, they differed in their distribution and generated sample similarity patterns similar to those represented by the whole community. The genera/lineages favored in either the eutrophic or oligotrophic waterways generally matched what is known about the lifestyles of these organisms. The urban estuary favored *Betaproteobacteria* genera including *Limnohabitans*, a genus defined by its fast-growth rates and copiotrophic lifestyle (Šimek et al., 2006; Jezbera et al., 2011), and *Rhodobacter*, a genus frequently abundant in near-shore eutrophic conditions, but less common in the pelagic low-nutrient freshwater environment (Imhoff, 2006; Newton et al., 2011a). In contrast, the lineages acI and LD12 were favored in oligotrophic Lake Michigan. Both of these lineages are characterized by slower-growth, small cell sizes, and predation avoidance or oligotroph life strategies (Newton et al., 2011a; Salcher et al., 2011b; Ghylin et al., 2014). These results suggest that even at fairly broad taxonomic characterization such as genus or phylogenetic lineage there may be conserved characteristics within some freshwater groups, which contribute to community assembly patterns across urban/trophic gradients.

### Within genus/lineage composition change

Recently, several studies have identified within-genus and with-species organism distribution patterns related to the biological and environmental properties of freshwater habitats. For example, it is now known that the ubiquitous freshwater bacterium *Polynucleobacter necessarius* subspecies *asymbioticus*, members of the genus *Limnohabitans*, and *Flavobacterium* each contain dozens of ribosomal gene sequence variants differentiated in their spatial and temporal distributions by lake characteristics such as pH, conductivity, and dissolved organic matter (Jezbera et al., 2011, 2013; Neuenschwander et al., 2015). Here we used an oligotyping approach to provide both high discriminatory power among closely related sequences (as low as one nucleotide) and to reduce the effects of sequencing errors (Eren et al., 2013a), so that we could better resolve distribution patterns within some of the most common freshwater bacterial genera/lineages. Despite the near ubiquity of the 18 examined freshwater genera/lineages, we observed the greatest community distinction between the urban estuary and Lake Michigan samples when using the higher organism discrimination provided by oligotyping. We also found that 8 of the 18 examined freshwater genera/lineages harbored both oligotypes that were favored in the urban estuary and oligotypes favored in Lake Michigan, including several instances where these opposite distribution patterns occurred among oligotypes with one or two nucleotide differences. It appears diversification is high within many of the ubiquitous freshwater bacterial genera and often includes organisms with distinct advantages over other closely related organisms in either eutrophic or oligotrophic waters. Together these results indicate that in addition to taxa augmentation, and common freshwater genus/lineage life strategy differences, a third mechanism, within-genus diversification, is driving community assemblage differences between the urban-influenced and Lake Michigan waters.

The combination of oligotyping and a habitat classification statistical approach also revealed a number of interesting trends among the common freshwater genera/lineages. The *Bacteroidetes* phylum, especially the genera *Flavobacterium, Fluviicola*, and *Sediminibacterium* had especially high oligotype richness, suggesting either the diversity of freshwater organisms associated with these genera is high or that a large number of urban-associated organisms belonging to these genera are delivered via city surface runoff and stormwater. *Flavobacterium* and *Sediminibacterium* had a large number of rare oligotypes, which supports the idea that many of these organisms are immigrants from the urban-environment. However, the *Flavobacterium* genus also contained a large number of oligotypes classified as urban-water specialists. The described diversity within this genus is immense and includes a number of fast-growing, opportunisitic species-like phylotypes (Neuenschwander et al., 2015) that are common in lotic systems (Read et al., 2015), which suggests these organisms should be common in many urban-influenced systems. Interestingly, the most abundant *Flavobacterium* oligotype was a Lake Michigan specialist and the only one of the 16 *Flavobacterium* oligotype specialists that was not urban-water associated.

A number of other genera/lineages were dominated by oligotypes assigned primarily to one of the environmental specialist categories. The commonly noted oligotroph clades acI-A, acI-B, and LD12 (Newton et al., 2011a) contained only Lake Michigan specialists. The genus *Fluviicola*, also contained a large number of Lake Michigan specialist oligotypes, but at this time relatively little is known about this genus (Salcher et al., 2011a). It is unlikely we over-classified oligotypes as specialists, as we chose a conservative criterion for classification (specialization *K* = 3∕4; Chazdon et al., 2011). We also found some groups had a high number of oligotypes classified as generalists (e.g., acI-A, *Fluviicola*). It may be that some common freshwater organisms are true euryoecious organisms, resulting in broadly abundant distributions. It is also likely many generalist classifications are the result of our inability to distinguish among organisms with short-read 16S rRNA gene technologies. Recent studies have shown that the 16S-23S internal transcribed spacer (ITS) region, a less conserved bacterial genomic region, was able to identify organism distribution patterns among lakes that were otherwise obscured when examining 16S rRNA gene data (Jezbera et al., 2013; Hahn et al., 2015). The combined results of this study and the previous studies using ITS-based sequence groupings, indicate that more narrowly-defined organismal approaches are necessary to further our understanding of the biogeography and ecology of the ubiquitous freshwater pelagic bacteria.

### Technical considerations

The data in our sequence-based analyses were derived from three different sequencing platforms: 454 V6, 454 V6V4, and illumina V6. The choice of gene amplification conditions and sequencing platform are known to influence the composition of the resultant sequence data (e.g., Wu et al., 2010; Schloss et al., 2011). We also observed an influence of sequencing conditions on our bacterial community composition data (see Supplementary Figure 1 and associated Results Section); however, this influence on the overall community composition and diversity was small in comparison to the influence of the primary environmental gradient examined. Also, in all cases, the dominant freshwater oligotypes were present across all three sequencing platforms (see Figure 5 for example), which suggested that although our analyses were influenced by the platform used, the differences did not manifest in the loss/gain of dominant freshwater groups. We agree with previous work that the use of a single sequencing platform gives the most robust cross-sample comparisons, but in the case of some meta-analyses, including this one, these data may not exist. Our data suggest that cross-platform comparisons of 16S rRNA gene data are feasible and can give meaningful results especially when care is taken to quality-control sequence output and strong environmental gradients are examined. We suspect that if a single sequencing platform had been used here, the within-habitat diversity estimates and community composition variation in our data would have decreased and therefore furthered the distinction between the communities in urban-influenced waterways and oligotrophic Lake Michigan.

## Conclusions

In our study system, water flows from the urban-impacted Milwaukee estuary into oligotrophic Lake Michigan, and with it, the estuary bacterial assemblage is continuously dispersed into the lake. Despite this direct connection, our examination of the bacterial communities across this environmental gradient revealed quite distinct assemblages. We found Lake Michigan harbors lower bacterial diversity than the urban-impacted estuary, shifts the dominance among common freshwater genera/lineages, and selects for what are likely unique species or populations within many of the common freshwater bacterial lineages. These data support the idea that the oligotrophic lake represents a strong selective force favoring a particular set of cosmopolitan freshwater taxa and largely prevents the successful dispersal of bacteria from the urban environment. It remains to be seen whether smaller but heavily urban-influenced lakes are more likely to contain persistent bacterial populations of urban origin. Either way, it is clear the environmental conditions in these urban waterways impact heavily the composition of the core freshwater community and increase the prevalence of bacteria that are not common to pelagic freshwaters.

The fact that many of the common freshwater genera/lineages harbored both “urban-estuary” and “Lake Michigan” specialists, further suggests the ubiquity of many common freshwater bacteria is a result of large-scale diversification within these groups (e.g., Jezbera et al., 2011; Hahn et al., 2015). Given the “island-like” nature of lakes across the globe and an ongoing desire to understand microbial diversification in natural systems, the study of within-genus or within-species genetic diversification of lake bacteria warrants further exploration. Whether or not urban waterways alter significantly the ecological function of these bacterial communities, select for genetic compositions or functional traits that are distinct from un-impacted surface waters, or contribute to the maintenance and/or proliferation of microorganisms that impact human health or well-being is yet to be determined. Further integration of the microbial components of urban landscapes is needed in the ongoing development of an ecological understanding and theory for urban areas.

## Funding

This work was funded by the National Institutes of Health grant R01-AI091829 and MMSD Contract M03029P10 to SM.

## Author contributions

RN and SM conceived the work and analyses. RN carried out the data collection and analyses. RN and SM wrote the paper.

### Conflict of interest statement

The authors declare that the research was conducted in the absence of any commercial or financial relationships that could be construed as a potential conflict of interest.

## Footnotes

^1^Standard Methods for the Examination of Water and Wastewater 20 th ed., 1998. American Public Health Association (APHA), 1015 15th Street, NW, Washington, DC 20005.
