# Supplementary material for: A unique assemblage of cosmopolitan freshwater bacteria and higher community diversity differentiate an urbanized estuary from oligotrophic Lake Michigan
Source: Front Microbiol. 2015 Sep 29;6:1028. doi: 10.3389/fmicb.2015.01028 (PMC4586452; doi:10.3389/fmicb.2015.01028)
Supplement: Supplementary file 2 [file Presentation1.PDF]

Supplementary Material

Differences in the bacterial community structure between urban-impacted waterways and oligotrophic Lake Michigan

Ryan J. Newton[1*] and Sandra L. McLellan[1]

[1]School of Freshwater Sciences, University of Wisconsin-Milwaukee, Milwaukee, WI, USA.

Running title: Urban impacts on bacterial communities

Keywords: bacterial community, freshwater, urban ecology, Lake Michigan, oligotyping, bacterioplankton

* Correspondence: Ryan J. Newton, School of Freshwater Sciences, Great Lakes Research Facility, University of Wisconsin-Milwaukee, 600 E. Greenfield Ave. Milwaukee, WI 53204 USA. Email. newtonr@uwm.edu

Supplementary Table 1. – Sample metadata table; See attached file.

Supplementary Table 2. Common freshwater oligotype habitat specialization classification[1,2]

Phylum	Genus/Lineage	Urban Estuary	Lake MI	Generalist	Rare	>97%[3] pairs	>96%[3] pairs
Actinobacteria	acI-A	-	3	8	8	-	-
	acI-B	-	5	1	1	-	-
	acI-C	1	1	-	1	1	1
	acTH1	-	1	1	1	-	-
	acSTL	-	-	1	-	-	-
	Aquiluna	2	1	-	21	-	1
Bacteroidetes	Arcicella	-	-	2	11	-	-
	Algoriphagus	-	-	1	8	-	-
	Flavobacterium	15	1	9	50	-	1
	Fluviicola	6	10	11	4	1	1
	Sediminibacterium	2	2	2	24	1	1
Proteobacteria	LD12	-	1	4	11	-	-
	Sphingopyxis	-	-	2	9	-	-
	Hydrogenophaga	8	-	5	12	-	-
	Limnohabitans	5	1	6	15	-	1
	Polynucleobacter	2	1	4	10	-	1
	Rhodobacter	8	-	5	11	-	-
Verrucomicrobia	Luteolibacter	2	2	6	6		
	Total	51	29	68	203	3	7

1. The number of oligotypes or oligotype pairs in each category is listed.

2. Oligotypes within each freshwater genus/lineage that preferentially associated with either the urban-influenced waterways or Lake Michigan, were identified via a multinomial species classification using the CLAM test (Chazdon et al., 2011) in the *vegan* R package (Oksanen et al., 2013). The CLAM test was performed on the sub-sampled dataset using an alpha value of 0.01 divided by the total number of oligotypes (n=351), a coverage limit of 30, and a specialization threshold of 0.75.

3. Indicates the number of oligotype pairs exhibiting opposite habitat specialization categorization and having either 1 nucleotide difference (>97%) or 2 nucleotide differences (>96%) in their V6 16S rRNA gene sequence representative.

References
Chazdon, R. L., Chao, A., Colwell, R. K., Lin, S. Y., Norden N., Letcher, S. G., et al. (2011). A novel statistical method for classifying habitat generalists and specialists. *Ecology* 92, 1332–1343. doi: 10.1890/10-1345.1

Oksanen, J., Blanchet, F. G., Kindt, R., Legendre, P., Minchin, P. R., O'Hara, R. B., et al. (2013). vegan: Community Ecology Package. R package version 2.0-8. Available at http://CRAN.R-project.org/package=vegan.

Supplementary Figure 1

Non-metric multidimensional scaling plot indicating the community composition relationships (Bray-Curtis similarity) between the sample sequence data generated with various sequencing platforms: V6 454 (blue), V6V4 454 (yellow), and V6 Illumina (green). Community composition is based on the grouping of sequences by taxonomic assignment to genus and compiled as the relative contribution of each taxon to the community.
