# Supplementary figures and images for: A unique assemblage of cosmopolitan freshwater bacteria and higher community diversity differentiate an urbanized estuary from oligotrophic Lake Michigan
Source: Front Microbiol. 2015 Sep 29;6:1028. doi: 10.3389/fmicb.2015.01028 (PMC4586452; doi:10.3389/fmicb.2015.01028)

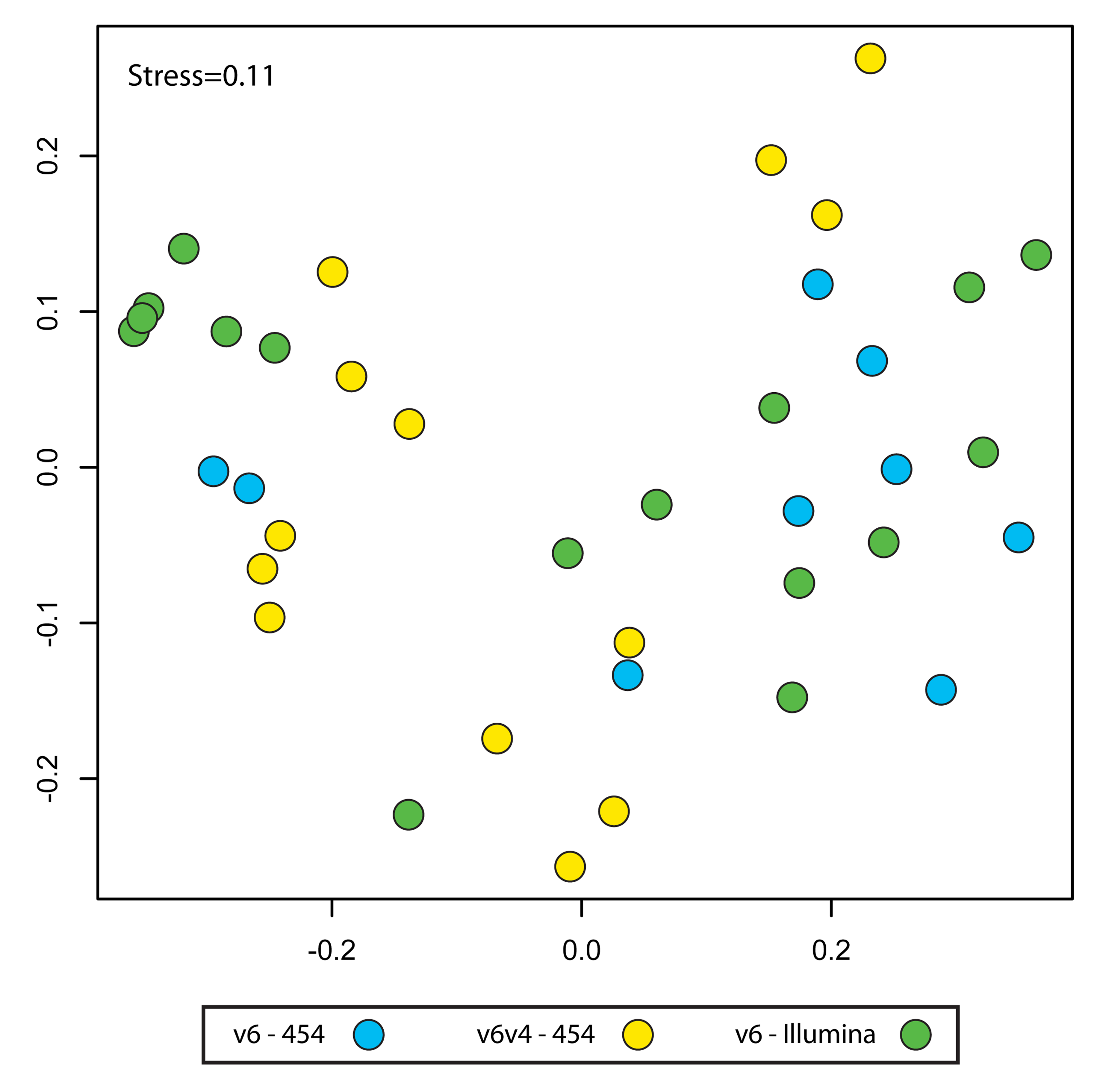

Supplement: Supplementary file 3 [file Image1.TIF]
